# Diffusion Tensor Imaging for Glioma Grading: Analysis of Fiber Density Index

**DOI:** 10.15412/J.BCN.03080102

**Published:** 2017-01

**Authors:** Fariba Davanian, Fariborz Faeghi, Sohrab Shahzadi, Zahra Farshifar

**Affiliations:** 1. Department of Radiology Technology, School of Allied Medical Sciences, Shahid Beheshti University of Medical Sciences, Tehran, Iran.; 2. Department of Neurosurgery, Shohada Tajrish Hospital, Shahid Beheshti University of Medical Sciences, Tehran, Iran.; 3. Department of Radiology Technology, School of Paramedical, Shiraz University of Medical Sciences, Shiraz, Iran.

**Keywords:** Diffusion tensor imaging, Neoplasm grading, Glioma, Fiber density index

## Abstract

**Introduction:**

The most common primary tumors of brain are gliomas and tumor grading is essential for designing proper treatment strategies. The gold standard choice to determine grade of glial tumor is biopsy which is an invasive method. The purpose of this study was to investigate the role of fiber density index (FDi) by means of diffusion tensor imaging (DTI) (as a noninvasive method) in glial tumor grading.

**Methods:**

A group of 20 patients with histologically confirmed diagnosis of gliomas were evaluated in this study. We used a 1.5 Tesla MR system (AVANTO; Siemens, Germany) with a standard head coil for scanning. Multidirectional diffusion weighted imaging (measured in 12 noncollinear directions), and T1 weighted nonenhanced were performed for all patients. We defined two regions of interest (ROIs); 1) White matter fibers near the tumor and 2) Similar fibers in the contralateral hemisphere.

**Results:**

FDi of the low-grade gliomas was higher than those of high-grade gliomas, which was significant (P=0.017). FDi ratio (ratio of fiber density in vicinity of the tumor to homologous fiber tracts in the contralateral hemisphere) is higher in low-grade than high-grade tumors, (P=0.05). In addition, we performed ROC (receiver operating characteristic) curve and the area under curve (AUC) was 0.813(P=0.013).

**Conclusion:**

Our findings prove significant difference in FDi near by low-grade and high-grade gliomas. Therefore, FDi values and ratios are helpful in glial tumor grading.

## 1. Introduction

The most common primary tumors of the central nervous system are gliomas (Chen, Shi, & Song, 2010; Kang et al., 2015). Glioma arises from the glial cells of the brain (Jin, Zhang, Yang, & Luo, 2015; Min, Niu, Rana, Ji, & Zhang, 2013). Despite improvement in prognosis for patients with low-grade gliomas, the prognosis is still poor for high-grade gliomas (Kayama, Kumabe, Tominaga, & Yoshimoto, 1996; Ma & Song, 2013). In this regard, tumor grading is essential for designing proper treatment strategies (Brat & Van Meir, 2004; Lee et al., 2008). The gold standard method for glial tumor grading is the biopsy which is an invasive method with its own problems and risks. Biopsy can lead to swelling or bleeding of the brain, infections, seizures, stroke, or coma. Sometimes tests on the sampled tissue are inconclusive and the procedure must be repeated. The resulting problems of biopsy depends on many variables like lesion properties (location, histology) and preoperative pharmacological therapy (corticosteroids, antiplatelet agents) (Sawin, Hitchon, Follett, & Torner, 1998). Intracranial hemorrhage is the most common side effect of brain biopsy and is associated with inpatient mortality and hospital disposition (Malone et al., 2015).

Based on recent studies, use of routine magnetic resonance imaging (MRI) in detection and assessment of lesions has been improved. However, MRI may underestimate tumor size and is not a reliable method in tumor grading, and that may cause mistake in treatment strategy (Johnson, Hunt, & Drayer, 1989; Watanabe, Tanaka, & Takeda, 1992).

Diffusion tensor imaging (DTI) is an MRI method that maps water molecules diffusion and is a totally noninvasive method. Because of different obstacles (like fibers and membranes), water molecules cannot freely diffuse. DTI by the aim of water molecule diffusion patterns, reveals the microscopic details about tissue architecture, including its fibers. Furthermore, DTI has become standard test for white matter disorders, because of its ability in defining abnormalities in fiber architecture (Hagmann et al., 2006). DTI is mainly used for study and treatment of neurological disorders.

Fiber density index (FDi) was introduced by Roberts and colleagues in 2015. They described FDi as density of fiber tract in glioblastoma tumors (Roberts, Liu, Kassner, Mori, & Guha, 2005). FDi is a quantitative description of fiber tracks in each pixel of the region of interest (ROI). In their study, 0.15 and 0.20 were reported as the best fractional anisotropy threshold to reconstruct fiber around a glioma. In this study, we choose a threshold of 0.20. While pathologic sampling, as a gold standard, is an invasive way, we perform prospective study to investigate the role of FDi by means of DTI (as a noninvasive method) in grading gliomas.

## 2. Methods

### 2.1. Study population

The imaging data of 20 patients (mean age±SD, 45.9±13.6 y), with histologically proven WHO low-grade (13 patients) and high-grade (7 patients) gliomas, were collected. About 45% (9 patients) were female (age range, 15–68 years; mean age, 48.3 years±15.9) and 55% (11 patients) were male (age range, 26–69 years; mean age, 43.9 years±11.89).

In this study, we considered MRI contraindications (claustrophobia, foreign body) and ethical issues (taking informed consents). After MRI examinations, biopsies were performed on all patients and according to the pathological result (as a gold standard), low-grade and high-grade gliomas were classified.

### 2.2. Magnetic resonance imaging and image analysis

In this study, we used a 1.5 Tesla MR system (AVANTO; Siemens, Germany) with a standard head coil for scanning. A diffusion-weighted echo-planar imaging sequence was performed (repetition time=8600 ms; echo time=107 ms; number of excitations=1). In 12 noncollinear directions, diffusion encoding gradient was performed. Diffusion weighting factors (b value) were b=1000 s/mm^2^ and b=0 s/mm^2^ (no diffusion gradient).

#### 2.2.1. Data processing

Spatial normalization and preprocessing on 20 DTI series and b0 image series were performed by using ExploreDTI (Leemans, Jeurissen, Sijbers, & Jones, 2009). Final processing was performed by the DTIStudio version 3.0.3 (Processing Tools and Environment for Diffusion Tensor Imaging–H. Jiang and Mori, Radiology Department, Johns Hopkins University, Baltimore, MD, USA) to measure FDi.

The fiber density index (FDi) is a quantitative measure of fiber tracks in each pixel. We should define the fractional anisotropy (FA) threshold and fiber angle threshold for reconstructing fibers (Mori, 2007). We described FA thresholds of 0.20 and angle of up to 70 degree to reconstruct fibers.

#### 2.2.2. ROI determination

**Figure 1:**
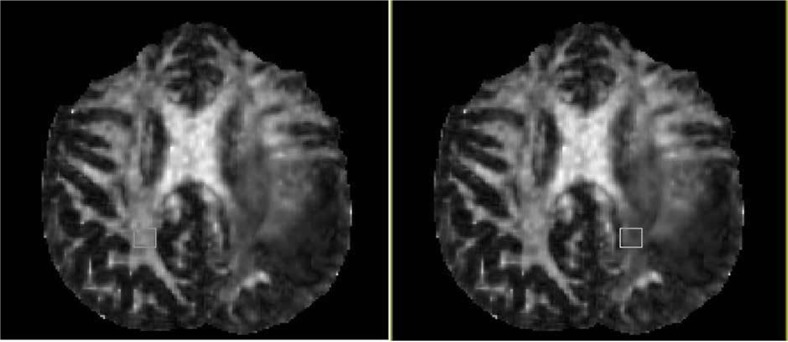
Determining regions of interest (ROI) in a 43-year-old man with high grade glioma, adjacent to white matter, and normal hemisphere ROI.

We defined 2 rectangular ROIs (10×10 pixels) for each patient: 1) The white matter adjacent to the tumor (in nearest identifiable fiber tracts to the gliomas); 2) The homologous fiber tracts to ROI 1 in the contralateral hemisphere (Figure 1). In this study, we used fractional anisotropy map for drawing ROIs to better define the anatomic relationship between tumor and adjacent fibers.

#### 2.2.3. Parameters measurement

Two parameters were measured for each patient: 1) FDi values were recorded for ROI 1 and 2; 2) FDi ratios: by dividing the measured FDi values in ROI 1 by those in ROI 2.

### 2.3. Statistical analysis

In this study, we used SPSS version 16.0 (Chicago, IL, USA) to analyze our data. We performed ROC (receiver operating characteristic) curve and used the AUC (area under curve) to evaluate the association between FDi and gliomas grading. Also, after normality assessment we performed the t test and Mann-Whitney test to investigate the correlation between our parameters and tumors grade.

## 3. Results

We used Mann-Whitney test to evaluate FDi values. Fiber density in the vicinity (FDit) of the low-grade gliomas (7.59±4.44) tended to be higher than those of the high-grade gliomas (2.87±2.09). We found this difference significant (P=0.017).

**Figure 2:**
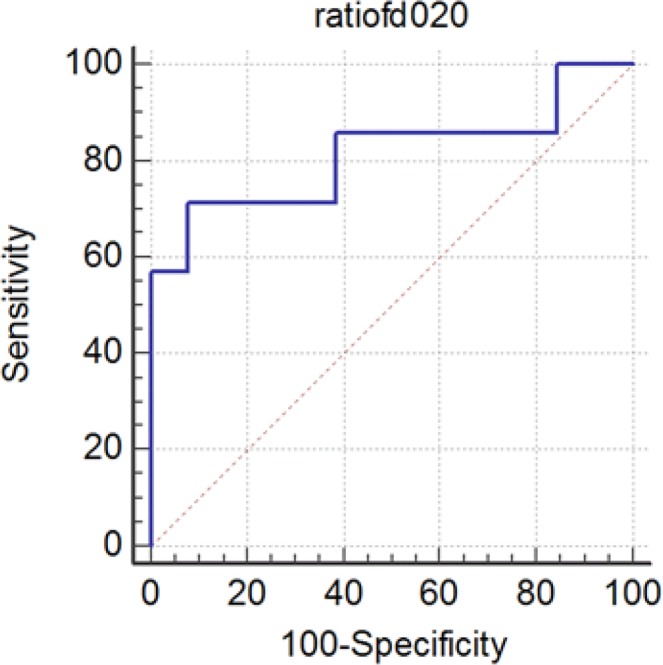
ROC curves for fiber density index ratio (FD_it/n_). ROC curve shape is close to square and indicates that the test has a high diagnostic value.

In addition, we used t test for FDi ratios (FDit/n) in high-and low-grade gliomas. As we expected, FDit/n is higher in low-grade gliomas (0.39±0.21) compared to high-grade (0.20±0.18) ones (P=0.05). Results of ROC curve are presented in Table 1 and Figure 2. Area under curve is 0.813 which is near 1, and that makes our test reliable.

**Table 1:** ROC curve test results on FD_it/n_ in glial tumor grading.

**P**	**AUC**	**95% CI**	**Specificity**	**95% CI**	**Sensitivity**	**Cutoff-value**	**Parameter**
0.013	0.813	64.0–99.8	92.31	29.0–96.3	71.43	0.2	FD_it/n_

Area Under Curve (AUC) was 0.8. The results were statistically significant (P<0.05).

## 4. Discussion

Our findings suggest that FDi values and ratios are higher in low-grade gliomas compared to high-grade ones. MRI basically depends on water molecules, and in this advance imaging method, DTI, we studied water molecule diffusion. Because of different obstacles (like fibers and membranes), water molecules cannot freely diffuse. In the presence and integrity of fibers (as an obstacle) water molecules diffusion pattern becomes more anisotropic. When fiber density or fiber packing and organizations get damaged and decrease, barriers for water molecules diffusion disappear and diffusion pattern become more isotropic (decrease of anisotropic diffusion of water molecules). In this way, DTI by the aim of water molecule diffusion patterns reveals the microscopic details about tissue architecture, including its fibers.

Based on that, our findings propose that the fiber tracts in the vicinity of low-grade gliomas are significantly preserved and well-organized, while peritumoral fiber tracts in high-grade gliomas are damaged, disorganized, and miss their integrity.

There are few published studies on FDi and gliomas so far. The most important study was done in 2005 by Roberts et al. who employed this parameter. They reported the reduction of this index in peritumoral white matter in comparison to the contralateral white matter (Roberts et al., 2005). We found significant FDi reduction, near high-grade gliomas compared to low-grade ones, and that would indicate fewer fiber paths traversing from the peritumoral ROI. In a similar study, Chen and colleagues suggested that the FDi values in white matter adjacent to the low-grade gliomas are higher than high-grade gliomas. Furthermore, they found that FDi ratios were significantly different between patients with high-grade and those with low-grade gliomas (Chen et al., 2010).

We expect more tumor infiltration and fiber disorganization in high-grade tumors. This study suggested that FDi is helpful to differentiate between the peritumoral tracts in high-grade gliomas (which are more destroyed) and low-grade gliomas.

In this study, we used FA-map (fractional anisotropy map), which can define (in a superior way) the anatomic relationship between tumor and adjacent fibers. It is also more standard than gray scale images (T1, T2) for this purpose. In this way, we better characterized peritumoral fibers and defined them in the contralateral hemisphere. Thus, our study has significant advantages over other studies. One cannot compare different fiber tracts with each other due to extensive variations. We employed ratios of FDi to overcome this problem. Our study limitation is the effect of the tumor, which may impact our findings. In this study, we investigated the role of FDi in glial tumor grading by means of DTI and found it helpful in glial tumor grading.
